# A Cyclic Vernier Two-Step TDC for High Input Range Time-of-Flight Sensor Using Startup Time Correction Technique

**DOI:** 10.3390/s18113948

**Published:** 2018-11-15

**Authors:** Van Nhan Nguyen, Duc Nha Duong, Yunmo Chung, Jong-Wook Lee

**Affiliations:** School of Electronics and Information, Information and Communication System-on-chip (SoC) Research Center, Kyung Hee University, Yongin 17104, Korea; nhannguyen@khu.ac.kr (V.N.N.); nhaduong@savarti.com (D.N.D.); chung@khu.ac.kr (Y.C.)

**Keywords:** time-to-digital converter, time-of-flight, input range, startup time, cyclic, Vernier, digitally controlled oscillator

## Abstract

Herein, we present a low-power cyclic Vernier two-step time-to-digital converter (TDC) that achieves a wide input range with good linearity. Since traditional approaches require a large area or high power to achieve an input range >300 ns, we solve this problem by proposing a simple yet efficient TDC suitable for time-of-flight (TOF) sensors. In previous studies using the cyclic structure, the effect of startup time on the linearity of the TDC is not described. Thus, the achievable linearity has been limited when the TDC is used for applications requiring a high input range. We solve this problem by using a simple yet effective technique to compensate. The proposed technique is realized using (1) digitally-controlled oscillators (DCOs) that have dual frequency control and matched startup time; (2) an alignment detector that performs startup time correction by proper timing control; and (3) a fully symmetric arbiter that precisely detects the instant of edge alignment. To achieve a fine resolution for the cyclic Vernier TDC, we design two closely-matched DCOs with dual frequency control. The alignment detector performs the critical task of cancelling startup time via timing control. The detector is delay-compensated by using a dummy to provide matched loading for the two DCOs. To enhance the detection speed under low power, a current-reuse approach is employed for the arbiter. The TDC is fabricated using a 0.18 μm complementary metal–oxide–semiconductor (CMOS) process in a compact chip area of 0.028 mm^2^. Measured results show a dynamic range of 355 ns and a resolution of 377 ps. When the result is applied for TOF sensing, it corresponds to a distance range of 53.2 m and a resolution of 5.65 cm. Over a relatively large input range, good linearity is achieved, which is indicated by a DNL of 0.28 LSB_rms_ and an INL of 0.96 LSB_rms_. The result corresponds to root mean square (RMS) error distance of 5.42 cm. The result is achieved by consuming a relatively low power of 0.65 mW.

## 1. Introduction

A time-to-digital converter (TDC) is widely used to quantize and digitize time interval information and regarded as one of the most important time sensor [1]. The time interval between pulse signals is used for various sensing applications, for example, altitude sensing [2], depth sensing [3], respiration rate sensing [4], biomedical image sensing [5,6], and distance sensing for navigation [7]. 

In reference [2], a TDC is integrated with the single-photon avalanche diodes (SPAD) to form the pixel of the sensor, which is used for the image sensor in short-range and for altimeters in the long-range applications. For image sensing applications, time-of-flight (TOF) measurements estimate the shape of an object by measuring the distance between the sensor pixels and the target object [3]. For biomedical sensing applications, the fluorescence lifetime image (FLIM) sensor uses TDCs to extract important information for identifying cell microstructure [5], which is not usually available from intensity image sensing. TDCs also have great utility in positron emission tomography (PET) image sensing [6]. In reference [7], a TDC is integrated into a distance sensing system for providing range information. 

Recently, challenging direct time-of-light (dTOF) applications have been reported using the SPAD equipped with high-performance TDCs. In reference [8], a technique to detect objects hidden from view and track them in real time is presented with a TDC having a 45.5 ps temporal resolution. In reference [9], a massively parallel FLIM microscope for live-cell FRET (Förster resonance energy transfer) imaging is presented with 52.5 +/− 0.7 ps resolution. In reference [10], a confocal non-line-of-sight (NLOS) imaging technique is presented to address the challenge of reconstructing the shape of hidden objects from extremely weak and multiply scattered light. Using a TDC having 4 ps resolution, this work achieves a precision up to 15.1 mm in 0.64 m distance. In reference [11], a depth and reflectivity imaging system with a 390 ps resolution single-photon camera is presented, which achieves high photon efficiency by exploiting both the transverse smoothness and longitudinal sparsity of natural scenes. Although these imaging system using dTOF are widely rely on TDCs, the depth imaging can also be realized using a time-gated image sensor, which achieves a relatively high fill-factor of 61% [12]. Imaging hidden objects are crucial in a diverse area of applications such as robot vision, medical diagnostics, remote sensing, and autonomous vehicles.

Thanks to the advantage of high speed and massive integration available with CMOS scaling, a modern sensor with monolithic integrated TDC has become a preferred approach [13,14]. Fast digital switching capability allows extracting fine time resolution and TDCs are realized using deep-submicron CMOS [15,16]. Because the operation of SPAD requires a high voltage, however, the imaging system is not usually realized in deep-submicron standard CMOS process. Another issue for integrated CMOS imaging system is that the SPAD pixel arrays usually suffer from a low fill-factor, which is attributed to the large area engaged with timing electronics. To overcome the drawback, techniques to integrate diffractive microlens arrays are presented for the SPAD fabricated using 0.35 μm high-voltage (HV) CMOS process [17,18]. In reference [19], the SPAD are realized in ultrathin-body silicon-on-insulator (SOI) process, which allows both frontside-illumination (FSI) backside-illumination (BSI) modes. The BSI mode allows imaging with a wide carrier region which is not blocked by metallization, achieving a virtually perfect fill factor. 

**Figure 1:**
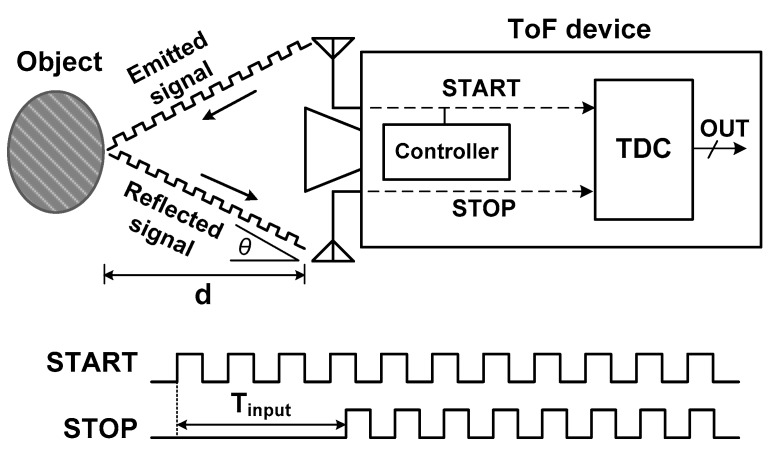
Operation of a time-of-flight (TOF) sensor. TDC: time-to-digital converter; STARTS: signal emission; STOP: detection.

For those sensing devices using the TDC, the operating principle is based on the TOF technique. Figure 1 shows the basic operation of a TOF sensing device. The sensor emits a signal with a known time reference and measures the reflected signal from an object. When the time difference *T*_input_ between signal emission (START) and detection (STOP) is measured, the distance *d* between the device and object is estimated using

(1) $$d=\frac{c\times T_{\mathrm{input}}}{2}\cos\left(\theta \right)\;$$

where *c* is the speed of the light and *θ* is the angle between the device and the object. If *d* is very large compared to the size of the device, *θ* is almost zero, leading to *d* = *c* × *T*_input_/2.

When a TOF sensor or a 3D camera is used to measure the distance or object’s surface depth, its range is directly related to the input range of the TDC [7]. Therefore, a high input range is desirable for many such sensing applications. For example, a high input range with fine resolution allows a camera to sense realistic shapes and surface textures of objects. Because long-time measurements are adversely affected by accumulated jitter and mismatch-related nonlinearity, realizing a sensor achieving a large input range is challenging [15]. Various strategies are reported to increase input range while achieving fine resolution, including two-dimensional (2-D) and three-dimensional (3-D) Vernier structure [16,20], cyclic Vernier [21], gated-Vernier oscillator [22], and a time-to-voltage converter (TVC) [23]. 

The Vernier TDC uses two delay lines to measure sub-gate delay or time delay smaller than the delay allowed by the process technology. Although this approach has the advantage of a simple implementation, the mismatch between the delay elements limits the achievable resolution. An improved approach involves replacing one of the delay lines with a delay latch chain [24]. This approach saves power and area in addition to reducing the mismatch of the conventional structure. However, the Vernier structure has an intrinsic limit for the achievable input range. This is because the range can only be extended by increasing the number of delay elements. To solve this problem, an interpolation technique is proposed [25], which is based on the Nutt method [26]. Another approach to reducing the length of the delay line involves extending the conversion dimension [16,20]. The 3D approach uses a delay line for the coarse step and a 2D Vernier plane for the fine step [20]. This approach achieves a moderate input range of 14 ns by using a relatively large area of 0.21 mm^2^. 

Another approach used to extend the performance of the TDC is using a time-to-voltage converter (TVC) followed by a successive approximation register (SAR) analog-to-digital converter (ADC) [23]. This approach converts the time interval into a voltage, and then quantizes the voltage by the ADC. In this approach, the design of a TVC with a high gain (for a full input range of the ADC) and linearity is challenging. Another drawback to this approach is the large area needed for the on-chip capacitors of the SAR ADC. 

The limit of the Vernier delay line for high input range can be solved by using a ring structure for time quantization. In this cyclic Vernier architecture, the two delay lines are replaced with two oscillators which have slightly different frequencies [21,22]. The work in [21] synthesizes a cyclic TDC using the standard cell library and achieves 5.5 ps resolution. The systemic mismatch of the oscillator delay, which is generated by automatic place-and-route, is handled by measuring and calibrating the buffer delay. The ring oscillator is, however, rather sensitive to the process-voltage-temperature (PVT) variation. To handle PVT variation, a gated-Vernier oscillator is proposed [22]. By the first-order noise shaping, the effect of PVT variation and layout mismatch in the oscillator is reduced; the achieved input range of 20 ns is not suitable for TOF applications that demand a large input range. 

To achieve a large input range, one can simply try to increase the number of bits of the counter. When the number of bits is increased, however, clock cycles integrate more jitter, which compromises linearity. As such, this approach is suboptimal as it suffers from degraded linearity and requires increased power. Another important design consideration for cyclic TDCs is the startup time, *T*_startup_, of an oscillator. The approach using the STOP signal to directly control the counter, which measures the number of oscillation cycles, cannot compensate for the delay caused by *T*_startup_, resulting in counting errors. For applications demanding a large input range, the error caused by *T*_startup_ has a significant impact on linearity [27].

Since traditional approaches [16,20,25], require a large area or high power to achieve a large input range, we solve this problem by using a simple yet efficient TDC suitable for TOF applications. In the previous studies using cyclic structure [21,25,28], the effect of *T*_startup_ on the linearity of the TDC is not investigated. Thus, the achievable linearity has been limited when the TDC is used for applications requiring an input range >300 ns. We solve this problem by using a technique to remove the *T*_startup_ error. 

To meet the demands of TOF applications which require the input range from sub-nanoseconds to hundreds of nanoseconds [7], herein, we present a low-power cyclic Vernier two-step TDC achieving good linearity over a wide input range. The TDC is realized using (1) digitally-controlled oscillators (DCOs) with dual frequency control and matched *T*_startup_; (2) a new alignment detector performing *T*_startup_ correction via proper timing control; and (3) a fully symmetric arbiter performing precise detection of the instant of edge alignment. To compensate for the delay caused by *T*_startup_, the alignment detector controls the counters with proper timing instead of using the STOP signal. By the proposed technique, the error caused by *T*_startup_ is effectively removed by measuring *T*_input_ from the time difference between two closely-matched DCOs. The experimental results show that the proposed *T*_startup_ correction achieves a good linearity over a relatively wide input range of 355 ns. When the result is applied to TOF range sensor, it corresponds to a detection range of 53.2 m and a resolution of 5.65 cm. Over the input range, good linearity is indicated by a differential nonlinearity (DNL) of 0.28 LSB_rms_ (root mean square of the least significant bit) and an integral nonlinearity (INL) of 0.96 LSB_rms_. When the INL is used to indicate the discrepancy between the measured and real distance [20], the corresponding error distance is about 5.42 cm.

## 2. Design

**Figure 2:**
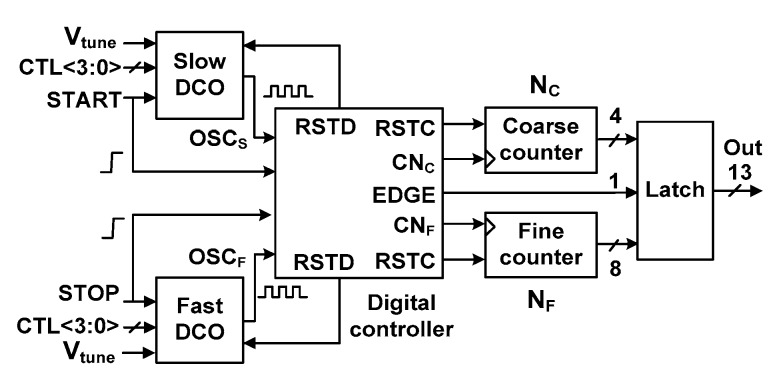
Block diagram of the proposed TDC. CN_C_ and CN_F_: coarse and fine counter clock signals; DCOs: digitally-controlled oscillators.

Figure 2 shows a block diagram of the proposed TDC. It consists of two DCOs, a digital controller, a coarse and a fine counter, and an output latch. The fast and slow DCOs generate outputs OSC_S_ and OSC_F_, respectively. The DCOs use dual control for fine frequency tuning, which is achieved by using digital control word CTL<3:0> and tuning voltage, *V*_tune_. The coarse and fine counter clock signals, CN_C_ and CN_F_, increment the count value during coarse and fine steps, respectively. An RSTD signal is used to reset the DCOs while the two counters are reset by the RSTC signal. In the cyclic Vernier structure, the fast DCO catches up with the slow DCO, resulting in edge alignment. The EDGE signal is used to distinguish two kinds (rising/rising and rising/falling) of the edge alignment. Using a 1-bit EDGE signal, 4-bit coarse counters and 8-bit fine counters, the TDC generates a 13-bit output. 

The TDC measures *T*_input_ between the rising edges of the START and STOP signals. The START signal enables the slow DCO that has a period of *T*_S_, and the coarse counter records the number of oscillation cycles, *N*_C_, in the coarse step. Then, the coarse time, *T*_coarse_, is obtained by multiplying *N*_C_ with *T*_S_. After a time delay of *T*_input_, the STOP signal arrives. It disables the coarse counter and enables the fast DCO that has a period of *T*_F_. The fine time *T*_fine_, which is less than *T*_S_, is measured by counting the number of cycles in the fine step, *N*_F_. Because *T*_S_ is slightly larger than *T*_F_, the time difference between the rising edges of the two DCOs is reduced by an amount *τ =* (*T*_S_ *− T*_F_) in every cycle. Here, *τ* is the resolution of the TDC. Then, *T*_input_ can be expressed as

(2) $$\begin{array}{ll} T_{\mathrm{input}} & =T_{\mathrm{stop}}-T_{\mathrm{start}}\\ & =T_{\mathrm{coarse}}+T_{\mathrm{fine}}=N_{C}\cdot T_{S}+\;N_{F}\cdot \left(T_{S}-\;T_{F}\right).\; \end{array}$$

**Figure 3:**
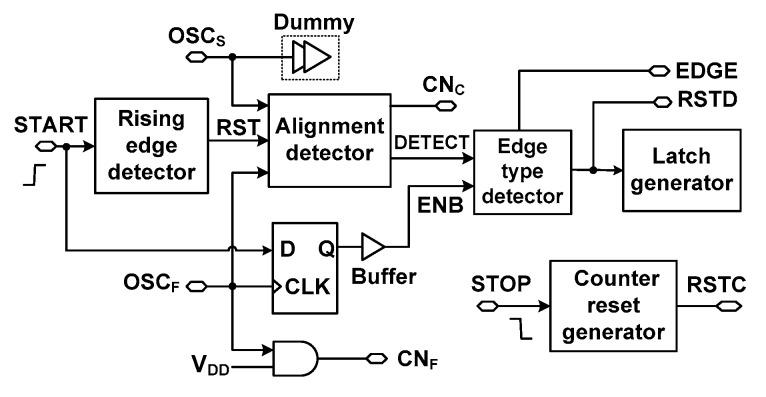
Block diagram of the digital controller.

**Figure 4:**
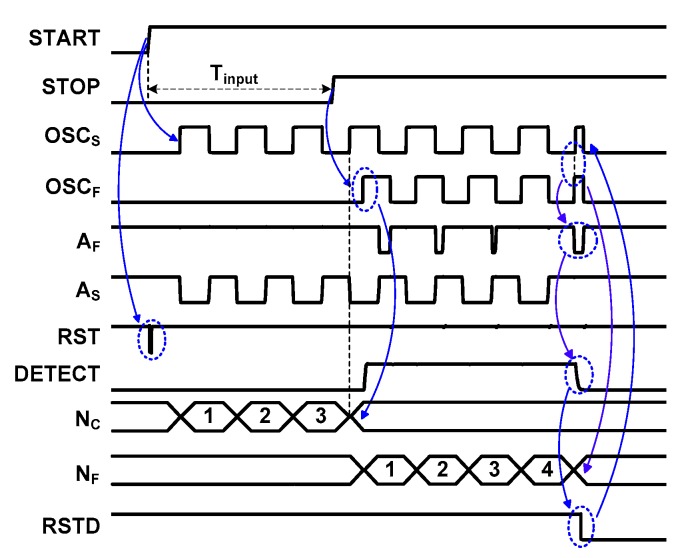
Timing waveform of the alignment detector.

To measure *T*_input_, the digital controller shown in Figure 3 generates a number of control signals. The controller includes a rising-edge detector, an alignment detector, an edge-type detector, a latch generator, and a counter reset generator. A brief description of these blocks is illustrated using the timing waveform shown in Figure 4. When the rising-edge detector is activated by the START signal, it generates a global reset, RST. Using the output of OSC_S_, the CN_C_ signal generated through the alignment detector increases *N*_C_. When the STOP signal arrives, it enables the fast DCO, which ends the coarse step. At this time, the alignment detector stops CN_C_ signal, and CN_F_ signal is generated by buffering OSC_F_ signal through a AND gate driven by a high voltage level V_DD_ (supply voltage), increasing N_F_. The STOP signal is also input to the counter reset generator for the RSTC signal, which is produced at the falling edge of the STOP signal. During the fine step, the phase of OSC_F_ eventually catches up to the phase of OSC_S_. The arbiter inside the alignment detector generates outputs A_S_ and A_F_. It captures the moment when the edges of OSC_S_ and OSC_F_ are aligned. At the moment of alignment, the DETECT signal from the alignment detector goes from high to low. Using the DETECT signal, the edge-type detector generates the RSTD signal, which stops the DCOs for power saving, and disables the CN_F_ signal. When the conversion is finished, the edge-type detector triggers the latch generator which stores the result in the coarse and fine counters. 

*A Startup Time Cancellation*

**Figure 5:**
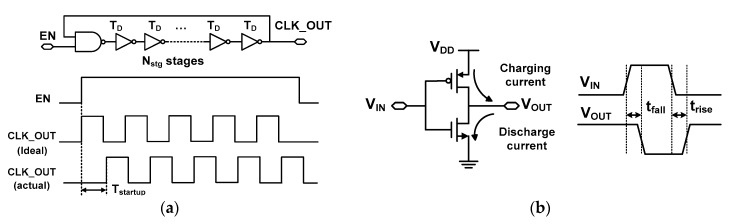
(**a**) Timing waveform of a ring oscillator. (**b**) Charging and discharging currents in an inverter.

Consider a ring oscillator having an odd number of inverter stages, *N*_stg_, as shown in Figure 5a. Ideally, when an enable signal EN is asserted, the oscillator would immediately generate the output CLK_OUT. In practice, each stage of the oscillator must accumulate some delay before producing an output. The propagation delay leads to startup time *T*_startup_. Assuming equal charging and discharging currents as shown in Figure 5b, the rise and fall times are approximately equal to *T*_D_ as *t*_rise_ = *t*_fall_ ≅ *T*_D_, where *T*_D_ is the average propagation delay of the inverter.

Assuming that each of the stages has the same characteristics, the cycle time (or period, *T*_cycle_) of the oscillator, which is the time it takes for the output to repeat its phase, can be expressed [29] as

(3) $$T_{\mathrm{cycle}}≅2N_{\mathrm{stg}}T_{D}\;$$

In the ring oscillator, *T*_startup_ is the signal propagation time after the EN signal is enabled. Neglecting delay in the logic gate, it can be expressed as the sum of delays as

(4) $$T_{\mathrm{startup}}≅N_{\mathrm{stg}}T_{D}=T_{\mathrm{cycle}}/2\;$$

*T*_startup_ is affected by various factors such as clock jitter, supply noise, and PVT variation. Nevertheless, Equation (4) shows that *T*_startup_ is relatively large, on the order of *T*_cycle_. For example, the slow DCO in this study runs at 43.1 MHz with *T*_S_ = 23.2 ns. In this case, *T*_startup_ ≅ *T*_cycle_/2 is 11.6 ns. 

**Figure 6:**
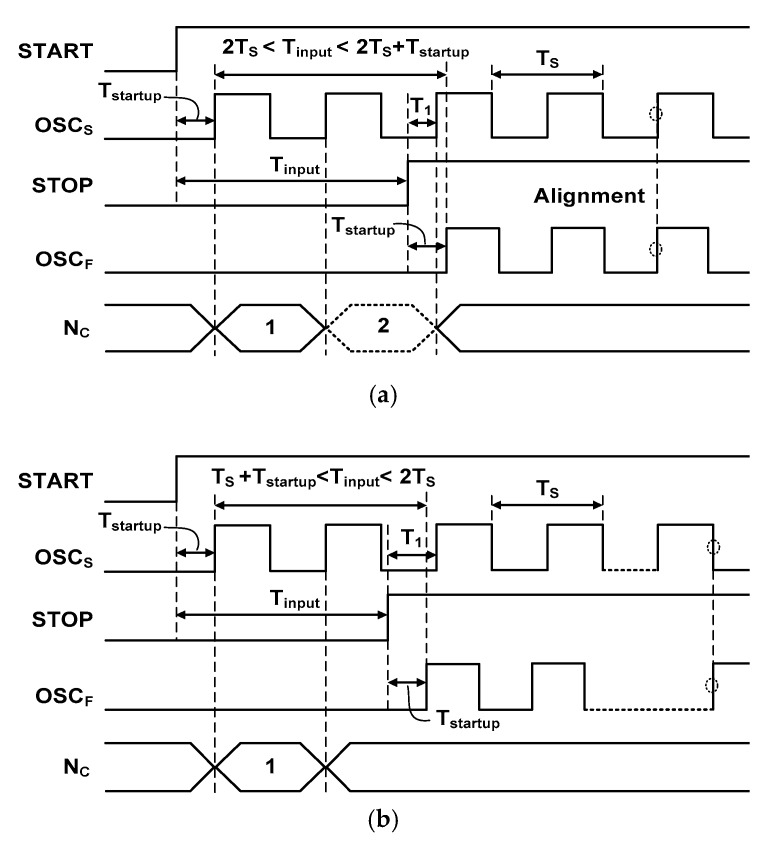
Waveform of two cases of the input time for a large *T*_startup_. (**a**) Incorrect counting by the startup time. (**b**) Correct counting.

To investigate the effect of *T*_startup_ on the linearity of the TDC, we consider the waveform as shown in Figure 6. It shows two cases for a relatively large *T*_startup_ *T*_cycle_/2. Here, we consider the period of coarse step with *T*_cycle_ = *T*_S_. Figure 6a shows the case when *T*_input_ is in the range of *k* × *T*_S_ *<* *T*_input_ *< k* × *T*_S_ + *T*_startup_ (*k* = 2 in this case). When the START signal initializes the counter, the cycle of the OSC_S_ is counted until the STOP signal disables the counter. In the case shown in Figure 6a, the counter generates an incorrect output of (*k* − 1) while the correct value is *k*. This error occurs because *T*_1_ (between the rising edge of STOP and the 3rd cycle of OSC_S_) is less than *T*_startup_. This error can be explained more generally as follows: after the START signal, the counter *has to wait* an amount of *T*_startup_ to measure an oscillation cycle. However, when the STOP is activated, the counting is immediately disabled. This error cannot be simply corrected by adding one to the coarse value because there is another case. Figure 6b shows the case of *j* × *T*_S_ + *T*_startup_ < *T*_input_ < (*j* + 1) × *T*_S_ (*j* = 1 in this case). When the oscillation cycle is counted between the START and STOP signals, it generates the correct value of *j*. This case of correct counting occurs as long as *T*_startup_ is less than *T*_1_. 

**Figure 7:**
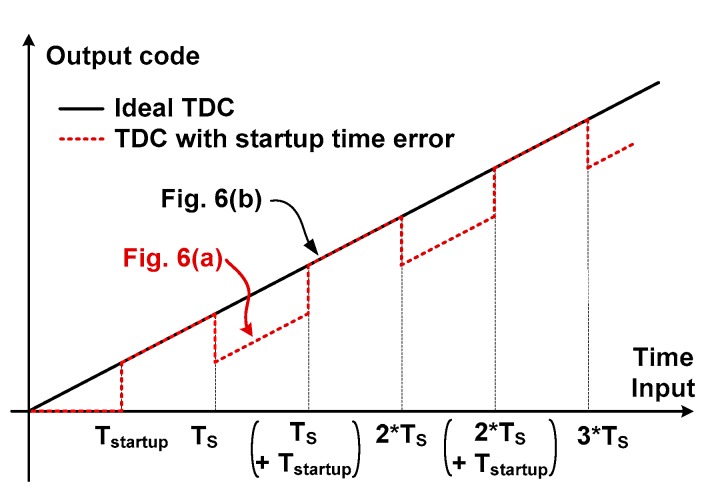
Input and output characteristics of the TDC for a large *T*_startup_.

Figure 7 shows the effect of *T*_startup_ on the transfer characteristic of the TDC. The error occurs in the region corresponding to the case of Figure 6a. The result shows that the output is periodically disturbed by the error. In the case when the STOP signal is used directly to stop OSC_F_, we note that the TDC does not generate the output when *T*_input_ *< T*_startup_. This is because OSC_S_ is not yet started and the STOP signal has already arrived. Therefore, there are none of any oscillations initiated in the two DCOs. The *T*_startup_ affects only the coarse step and generates the coarse counting error. Therefore, the influence of *T*_startup_ can be ignored when *T*_input_ is small, for example, the error does not occur for the case of *T*_startup_ < *T*_input_ < *T*_S_ as shown in Figure 7. When *T*_input_ is small, the TDC skips the coarse step and operates only in the fine step. Although OSC_F_ is delayed by *T*_startup_, we note that a similar delay occurs in the OSC_S_. In this work, we extract *T*_input_ from the first incoming edges between OSC_S_ and OSC_F_. Therefore, *T*_startup_ does not generate an error in the fine step. In the case when *T*_input_ is in a small range, for example, 2*T*_S_ < *T*_input_ < 2*T*_S_ + *T*_startup_ as shown in Figure 6a, the coarse error can be considered as an offset which can be removed by post-processing. When *T*_input_ is large, however, the error cannot be considered as an offset. Because the range of the *T*_input_ is unknown prior to the measurement, calibration of this nonlinearity is rather complicated.

The above result indicates that the effect of *T*_startup_ on the TDC’s linearity may be mitigated by reducing *T*_startup_. By modifying the logic gate where the EN signal is applied, we can indeed reduce *T*_startup_; when the logic gate for the EN signal in Figure 5a is moved to the last stage of the oscillator, it can be shown that *T*_startup_ is inversely proportional to *N*_stg_ as *T*_startup_ ≅ *T*_cycle_/(2*N*_stg_) [29]. By increasing *N*_stg_, *T*_startup_ can be reduced; this is achieved with increased power, accumulated jitter, and low speed (*T*_cycle_ ≅ 2*N*_stg_*T*_D_).

**Figure 8:**
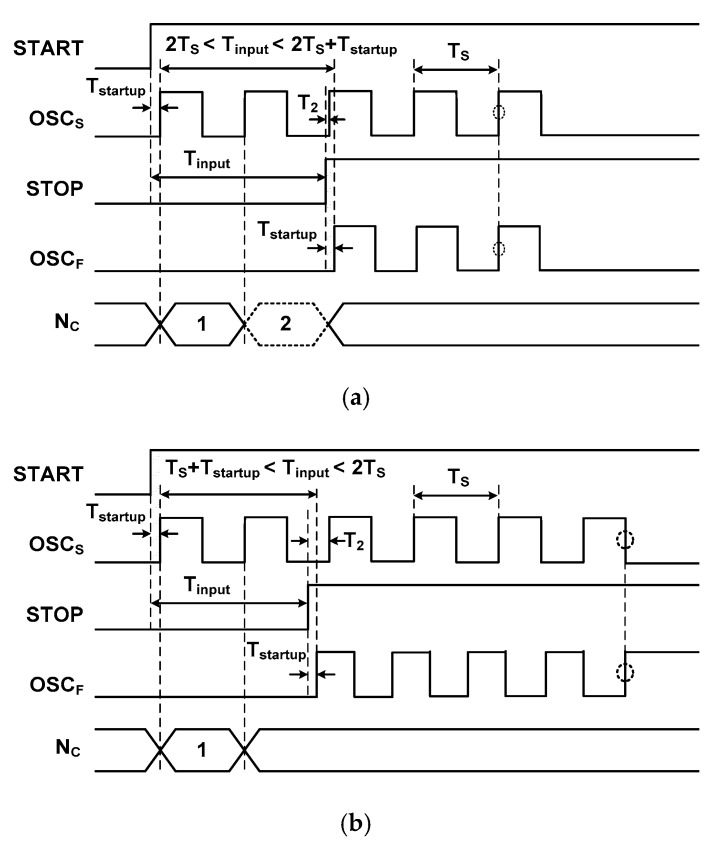
Waveform of two cases of the input time for a relatively small *T*_startup_. (**a**) Incorrect counting by the startup time. (**b**) Correct counting.

Figure 8 shows two cases of waveforms for a relatively small *T*_startup_. The result is shown for *T*_startup_ ≅ *T*_S_/(2*N*_stg_) with *N*_stg_ = 5. Figure 8a shows the case of *k* × *T*_S_ *<* *T*_input_ *< k* × *T*_S_ + *T*_startup_ (*k* = 2 in this case). Here, the STOP signal is activated before the 2nd cycle of OSC_S_ is finished and the counted value is (*k* − 1), which is incorrect. The error is generated because *T*_2_ (the time between STOP and the rising edge of 3rd OSC_S_) is smaller than *T*_startup_, and OSC_S_ is delayed by *T*_startup_. Figure 8b shows a case in which the STOP signal is activated before the 2nd cycle of OSC_S_ is finished, which counts the correct value. In this case, *T*_2_ is larger than *T*_startup_, and the small *T*_startup_ time does not affect the counting result. These results are similar to those shown in Figure 7, except for the reduced width of the error region, showing that even when *T*_startup_ is reduced, incorrect counting still occurs in some cases. Therefore, the output is necessarily disturbed by a constant offset in the transfer characteristic. 

The counting error caused by *T*_startup_ is related to the method used to detect the moment when the coarse step ends. In the actual TDC, the START signal for the coarse step is delayed by an amount of *T*_startup_. In the case when the STOP signal is used to disable the coarse step, there is no compensation for the delay caused by *T*_startup_. Therefore, the conventional approach of using the STOP signal to end the coarse step can generate the counting error. In summary, this method generates an error because OSC_S_ is used to *directly* clock the counter.

To achieve good linearity over a wide input range, we propose a startup time correction technique. The proposed technique is based on the idea of using two DCOs with matched *T*_startup_. In this system, *T*_input_ is equal to the time difference between OSC_S_ and OSC_F_ instead of between the START and STOP signals. In more specifically, CN_C_ is generated from OSC_S_. Before OSC_F_ is activated in the coarse step, the number of OSC_S_ cycles is counted to generate *N*_C_. When OSC_F_ is activated in the fine step, it controls the latch to stop generating *N*_C_. In this way, *N*_C_ is generated *indirectly* from OSC_S_, not from the delayed rising edge of OSC_S_. The proposed solution is implemented using the alignment detector as explained in the next section. 

*B. Alignment Detector and Arbiter*

**Figure 9:**
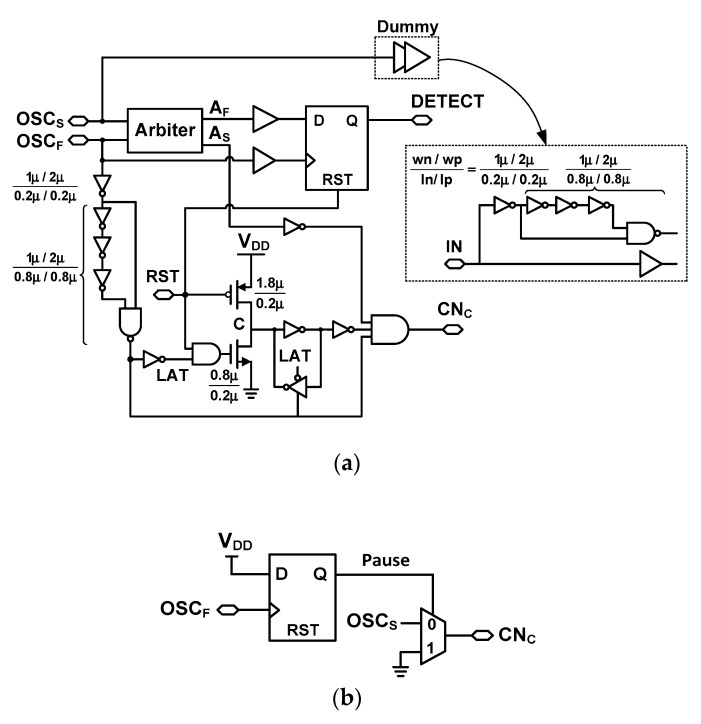
(**a**) Schematic of the proposed alignment detector, (**b**) alternative approach to generate CN_C_.

Figure 9a shows the schematic of the alignment detector. It performs two critical tasks: (1) detecting the edge alignment of two DCOs and (2) controlling the counter timing for startup time correction (explained above). When the conversion is started, an active-low reset signal RST changes the charging node ‘C’ to high and enables the logic gate that follows. In the coarse step, OSC_S_ is enabled. The arbiter receives the OSC_S_ signal and its output A_S_ goes through another inverter to generate CN_C_. The CN_C_ signal is a delayed and gated version of OSC_S_, which is used to increment the coarse counter. It is generated through the arbiter to immediately stop the counting when the edge alignment occurs. During the coarse step, OSC_F_ is inactive and the latch control signal LAT is low at this time. Thanks to the cross-coupled inverters used for the latch, the node ‘C’ is kept at a high level during the coarse step. The reason why we use the latch is to make sure that the NAND logic used for gating the CN_C_ is enabled even node ‘C’ is being discharged by leakage current. 

The coarse step is finished when OSC_F_ is activated. It generates a short pulse for the LAT signal, which pulls down the node ‘C’ and disables the logic gate for CN_C_. And the TDC enters the fine step. In the fine step, OSC_F_ catches OSC_S_ after some number of cycles. The alignment moment is detected by an arbiter followed by a D flip-flop (D-F/F), which generates the DETECT signal. The latch is added because the output of the arbiter is level sensitive.

We note that the activation and de-activation of CN_C_ are performed by the two DCOs having closely matched *T*_startup_; the time between START and OSC_S_ is closely matched to the one between STOP and OSC_F_. By using OSC_F_ to control CN_C_, the delay of *T*_startup_ does not cause an error in the coarse counter. In comparison, the conventional approach using the STOP signal for counter control does not consider *T*_startup_ and can result in incorrect counting as shown in Figure 7. 

We may consider using OSC_S_ as the coarse counting clock and utilizing the first rising edge of OSC_F_ to directly stop the coarse counting. This approach looks intuitive, however, it can create unbalanced load in the OSC_S_ and OSC_F_ paths. In addition, the implementation of the counter control signal for CN_C_ requires a *pause* function instead of a simple reset. When OSC_F_ arrives, it should stop the coarse counter to end the coarse step. At this time, we note that its counted value should be preserved for TDC’s output. This is because OSC_S_ still operates during the fine step until coincidence with OSC_F_ is detected. One example circuit realizing the pause function is shown in Figure 9b. This approach has the advantage of simple logic and generates a small delay in stopping the CN_C_ signal. Because of the setup time of the D-F/F, however, this approach may generate an unexpected counting error. This is in particular problematic when *T*_input_ is close to an integer multiple of OSC_S_ period. Another issue of using the simple logic is that the information available from the output A_S_ of the arbiter is wasted; it can be used to stop the coarse counting without causing additional loading to the DCOs.

**Figure 10:**
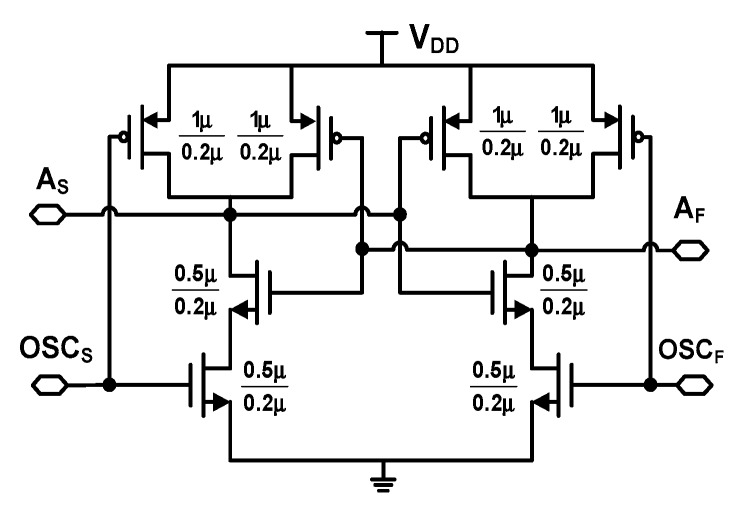
Schematic of the arbiter.

To achieve the proposed startup time correction, a good balance between the two outputs of the DCO is necessary. The arbiter itself has an inherently symmetric structure (see Figure 10). Therefore, we consider balanced loading at the two inputs of the arbiter. If the loading at the two ports is different, there can be a mismatch in the transient time of the two DCOs, which increases the probability of incorrectly detecting alignment. To achieve balanced loading, we insert a dummy at the output of OSC_S_. The size of the dummy is determined by performing extensive Monte-Carlo simulations that consider both local and global process variation. In this way, we are able to mitigate the load mismatch at the two outputs of OSC_S_ and OSC_F_. The upper limit to the estimated value of *T*_startup_ is *T*_cycle_/2 as indicated by Equation (4). In the case when the startup times of OSC_S_, (*T*_startup,S_) and OSC_F_ (*T*_startup,F_) are not equal, the difference can be approximated to half the resolution *τ* as

(5) $$T_{\mathrm{startup},S}-T_{\mathrm{startup},F}≅(T_{S}-T_{F})/2=\tau /2\;$$

Fortunately, the difference is an offset error with a mean value of *τ*/2, which can be easily removed by post-processing. We consider this post-processing because there can exist the mismatch caused by environmental noise and random jitter even when *T*_startup_ has been matched well in the simulated conditions.

Figure 10 shows the schematic of the arbiter. The arbiter is adapted from the previous work [6], and modified to reuse current for increasing the transconductance and speed. Similar to the arbiter [6], which keeps the output in good balance during the reset phase, the output of the proposed arbiter is also reset when both inputs are high. Circuit simulations show that the average current of the proposed arbiter is 16 μA at a supply voltage *V*_DD_ = 1.8 V. In comparison, the current is 53 μA for the arbiter [6], in which the high current is attributed to leakage current occurring when the two inputs are high and the outputs are in the middle. The proposed arbiter achieves a delay time from input to output of about 100 ps which is two times smaller than that of the previous work [6]. Compared with the conventional D-F/F and dynamic (true single-phase clocking-based) D-F/F, the proposed arbiter achieves better load balance. This is because the arbiter maintains a symmetric current steering path between two inputs while the D-F/F has different loads for the clock and data paths. 

Although the arbiter has a symmetric structure, an offset time, *t*_offset_, occurs due to the mismatch of device parameters, routing parasitics, and unbalanced loading. When the alignment of two DCOs occurs, the edge of OSC_F_ leads/lags that of OSC_S_ by the time difference *δ*. In the case of *δ* > *t*_offset_, the difference between the input and the estimated value is *τ* − *δ* which is smaller than the resolution *τ* of the TDC. In the case of *δ* < *t*_offset_, the fine step counts one more cycle and the error increases to 2*τ* − *δ*. By careful symmetric layout, we reduce *t*_offset_ < 2 ps, which is quite small when compared to the range of the TDC. 

*C. Digitally Controlled Oscillator*

**Figure 11:**
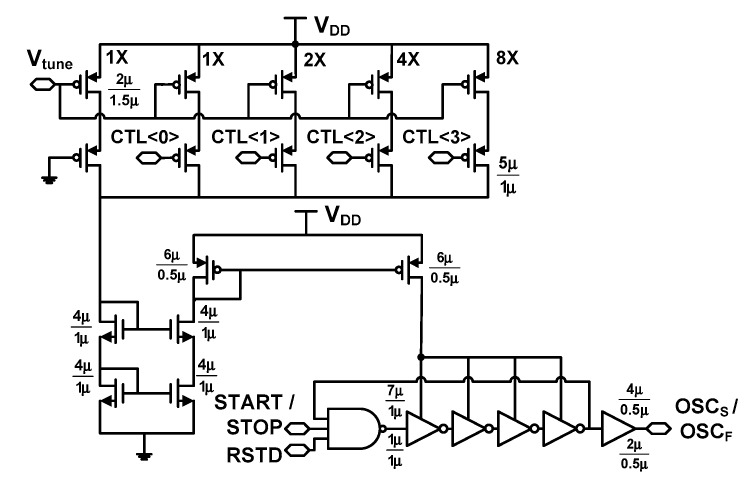
Schematic of the DCO.

Because the resolution of the cyclic Vernier TDC is determined by the frequency difference between the two DCOs, fine frequency control is necessary. In the previous study [6], there is no frequency tuning mechanism except the gating function. Our previous work included a DCO with a single control for frequency tuning [27]. In this work, we propose a DCO with dual frequency control. Figure 11 shows the schematic of the DCO. The oscillation is enabled by START and STOP signals for the slow and fast DCOs, respectively. The layout of this system is carefully designed to enhance matching between the two DCOs; the inverter stages of two DCOs are placed in an interdigitated manner and the binary-weighted current mirrors are designed using the common-centroid layout with a dummy. The dual-frequency control is implemented using four-bit control word CTL<3:0> and a tuning voltage *V*_tune_ applied to the current mirrors globally controls the amount of the current to the DCO. The CTL<3:0> adjusts the fine current via the digital-to-analog converter that acts as the discrete frequency controller for the DCO. The frequency *f*_DCO_ of the DCO can be expressed as

(6) $$f_{\mathrm{DCO}}=\frac{1}{2\times \left[T_{D,\mathrm{NAND}}+(N_{\mathrm{stg}}-1)T_{D,\mathrm{INV}}\right]}\;$$

where *T*_D,NAND_ and *T*_D,INV_ is the delay of the NAND gate and the inverter, respectively. When *V*_tune_ and CTL<3:0> are applied, only *T*_D,INV_ is varied while *T*_D,NAND_ is kept constant. Even though *T*_D,NAND_ is not controlled, it does not have much impact on the precise quantization of *f*_DCO_. This is because we are able to achieve fine-tuning of *f*_DCO_ using *V*_tune_. By varying *T*_D,INV_, the proposed dual frequency control method achieves frequency tuning in 20 kHz steps over a 4.8 MHz range. 

The RSTD is a reset signal for the DCO. When the conversion is finished, it stops the DCO to reduce power consumption. After the conversion, the counter is reset as is the accumulated jitter. This observation suggests that the error can be reduced by increasing the operating frequency of the DCO. However, the high operating frequency is accompanied by increased jitter. With this tradeoff and power consumption in mind, we choose the frequency of two DCOs to be 43.1 and 43.8 MHz. This corresponds to the resolution *τ* = 371 ps. 

*D. Edge-Type Detector*

**Figure 12:**
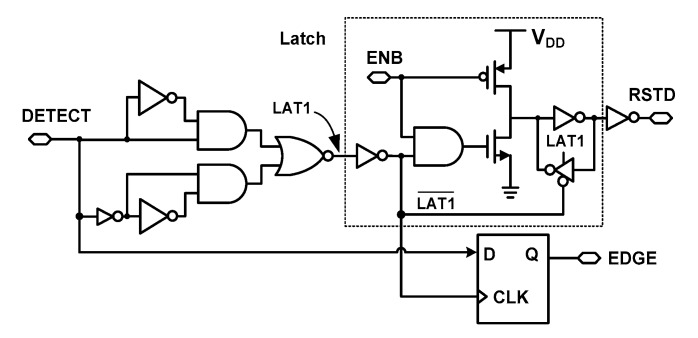
Schematic of the edge-type detector.

Figure 12 shows the schematic of the edge-type detector. The detector receives inputs DETECT and ENB and generates the EDGE and RSTD signals. The detector includes a D-F/F and a latch. The EDGE is generated through the D-F/F. The RSTD signal is generated through the latch which is controlled by the DETECT signal. This approach is used to ensure proper detection of edge alignment of the two DCOs. There are two transitions of DETECT signal (see Figure 4). The first transition occurs when OSC_F_ is starting and sets the DETECT signal to high. DETECT then transitions to low when alignment occurs. To prevent detecting a false alignment, the ENB signal is used to discard the first transition of the DETECT signal. 

The ENB signal is generated by the D-F/F in the digital controller (see Figure 3). Before the START signal, the low level of ENB keeps the RSTD at a high level. After the START signal, ENB is kept high. By the second transition of the DETECT signal, which indicates the end of conversion, the active-low RSTD signal is generated. This keeps the starting node of DCO high, thus freezing the oscillation, and it reduces power consumption by stopping the DCO. 

**Figure 13:**
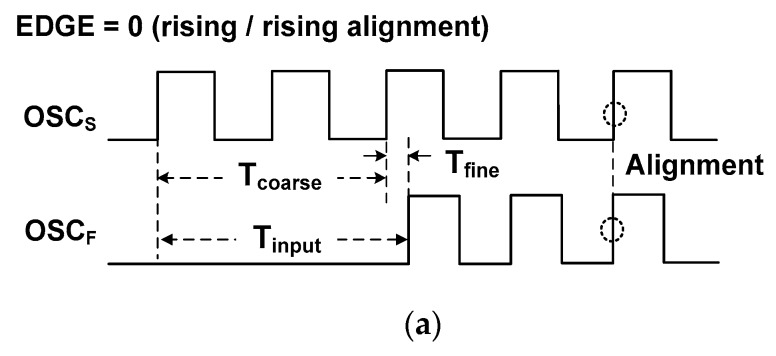

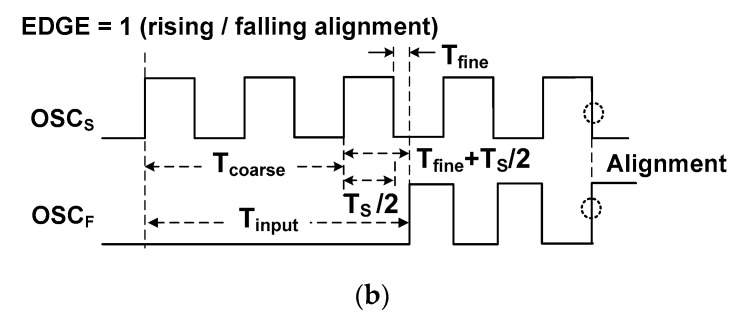
Waveform for two cases of EDGE bit. (**a**) Rising/rising alignment, (**b**) rising/falling alignment.

The EDGE signal indicates the type of edge alignment. In the case when both OSC_S_ and OSC_F_ are rising at the moment of alignment, as shown in Figure 13a, the DETECT signal goes from high to low generating EDGE = 0. This case corresponds to *T*_fine_ < *T*_S_/2. In the case of a rising/falling alignment, as shown in Figure 13b, the DETECT signal goes from low to high generating EDGE = 1. In this case, the duration of the fine step can be greater than half of a period. To handle this issue, we add *T*_S_/2 when EDGE = 1 so that the fine counter can measure a duration smaller than *T*_S_/2. In this way, the size of the fine counter is reduced by detecting both alignments. In addition, this strategy reduces power and increases conversion speed. Because of the operating sequence of the alignment detector in which OCS_F_ stops the CN_C_ signal (see Figure 9), CN_C_ is activated one more time. Since this occurs in every conversion, it can be considered as an offset to *N*_C_. Accounting for the offset and the status of the EDGE signal, the estimated time input $T_{input}^{*}$ is obtained using

(7) $$T_{input}^{*}=(N_{C}-1)\cdot T_{S}+\;\mathrm{EDGE}\cdot (T_{S}/2)+N_{F}\cdot \left(T_{S}-\;T_{F}\right).$$

## 3. Measured Results

**Figure 14:**
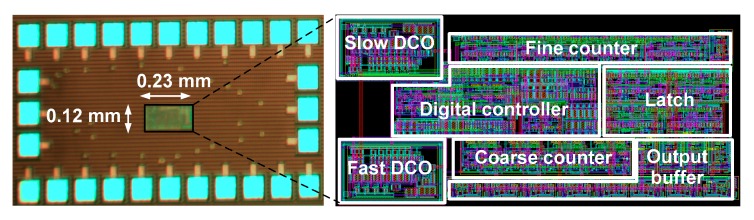
Chip microphotograph of the TDC.

**Figure 15:**
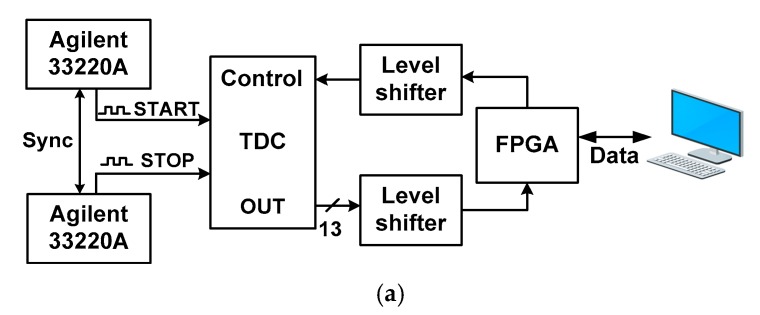

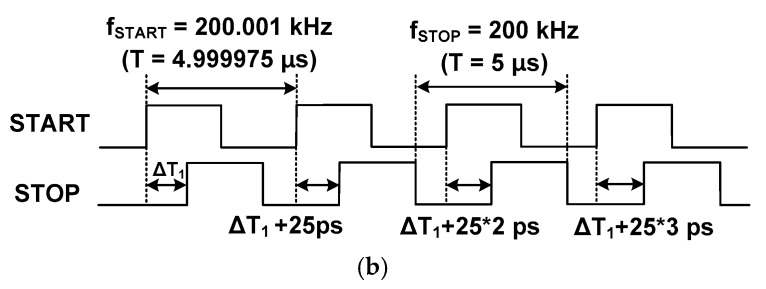
(**a**) Measurement setup, (**b**) time sweep method.

Figure 14 shows the chip microphotograph of the fabricated TDC with core layout. The size of the core is 0.028 mm^2^. Figure 15a shows the measurement setup for the TDC. Before characterizing the TDC, the DCOs are operated in a free-running mode to determine and calibrate the oscillation frequencies. Two function generators (Agilent 33220A) are used to create input signals (START and STOP). To reduce the number of I/O pads, a parallel to serial converter is included in the chip. An field-programmable gate array (FPGA) board is used to collect and convert the serial output to 13-bit data. Two level shifters are used to interface between the FPGA and the TDC. Finally, the data is transferred to a computer for further processing.

Characterizing the TDC is challenging, especially under the time sweep condition, because it requires precise control of time differences over a relatively long period. In this work, we use function generators to provide the START and STOP signals with slightly different frequencies. When the two signals are synchronized with a 1 Hz frequency difference, this method allows for increasing *T*_input_ in steps of 25 ps, as shown in Figure 15b. Thanks to the synchronization feature of the instrument, we precisely configure the time difference between two signals with a standard deviation of about 200 ps. Because the jitter from the equipment does not allow ideal time step, it does affect the test results. To handle this issue, we perform a number of measurements and take the average. In this way, the error caused by the jitter from the equipment can be averaged out. When combined with nonlinearity caused by mismatch, the linearity is further affected because more variation is added. Even with this nonlinearity, our measured result shows that the TDC achieves a relatively good linearity using the proposed approach (see Table 1).

**Figure 16:**
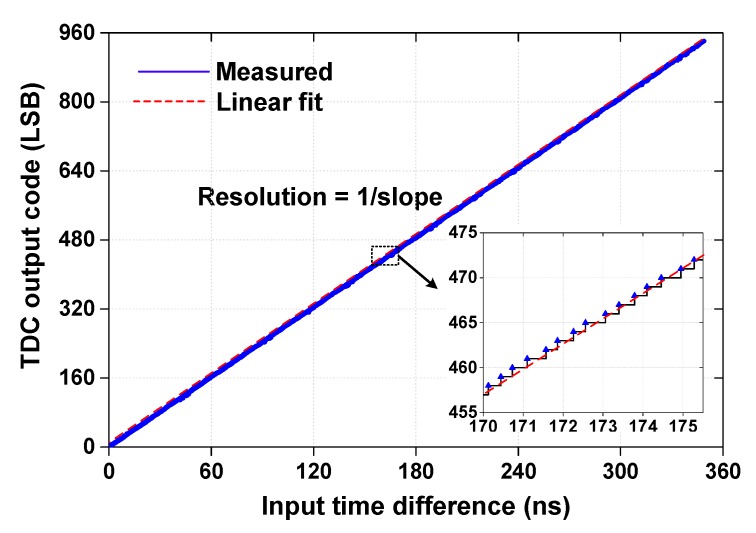
Measured transfer characteristic of the TDC.

Figure 16 shows the measured transfer characteristic of the TDC obtained by taking an average of eight measurements. Each measurement includes 14,400 steps with 25 ps increment. The result shows a relatively large input range of 355 ns. The maximum coarse and fine times of the proposed TDC are $(2^{N_{C}}-1+0.5)\times T_{S}$ and $T_{S}/2,$ respectively. The ‘−1’ term is used to correct the offset in the coarse clock; CN_C_ is activated one more time before OSC_F_ stops CN_C_ signal. The ‘+0.5’ term is added in consideration of the EDGE bit. The calculated result obtained using this expression is 371 ns which agree with the measured range. The inset of Figure 16 shows a magnified view of a portion of the curve. The resolution of a TDC is defined as the minimum time interval that a TDC can resolve [30], and it can be estimated as the reciprocal of the slope [6]. From the linear fitting curve of the data in Figure 16, we obtain a resolution of 377 ps, which agrees with the calculated resolution of 371 ps. The relatively low resolution is limited by the frequency difference of the two DCOs, which operate at a low speed for small power consumption; the result is still suitable for a long-range TOF application. When the result is used for range sensing, it corresponds to a detection range of 53.2 m and a resolution of 5.65 cm. 

System level simulations of the TDC show that the power consumption depends on *T*_input_; a longer fine step requires extended DCO oscillation time before the alignment event occurs, thus, consuming more power. When we take an average value for the maximum *T*_input_ = 355 ns, the TDC consumes 0.65 mW which is a total power (active and standby power). The latency also depends on the *T*_input_; it increases with each extended coarse cycle of *T*_input_. When the maximum range is covered, it requires the longest conversion time and in this case, the conversion rate is 0.67 MS/s.

**Figure 17:**
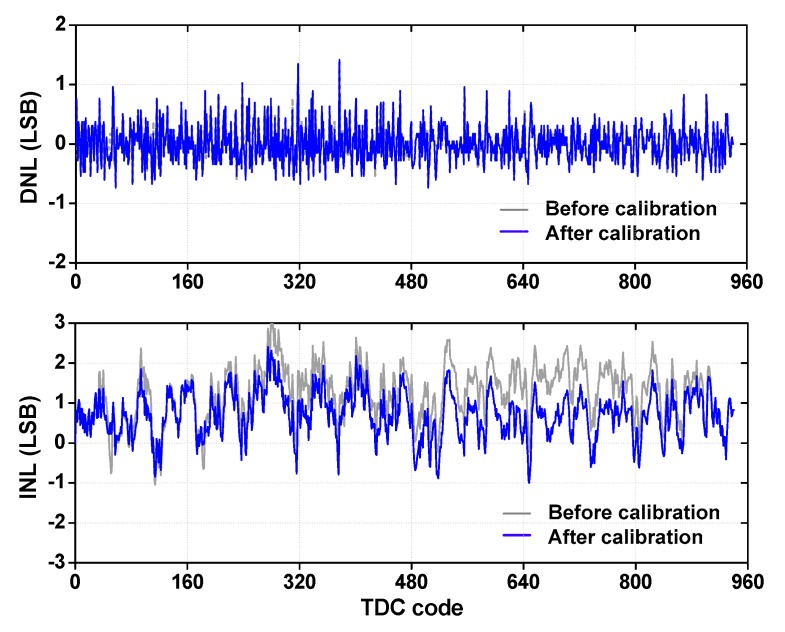
Measured differential nonlinearity (DNL) and integral nonlinearity (INL) of the TDC.

We obtain DNL and INL using code density method [6]. The size of the bin is 15 and the maximum code is 941 LSB. Figure 17 shows the result before and after calibration. The calibration is required because the static test is swept over a relatively long *T*_input_ and the input signals can be corrupted by the jitter from the equipment. To deal with this issue, we perform calibration by removing incorrect codes by checking consistency with the previous and subsequent codes. Using this method, we calibrate out 349 codes (2.4%) which are out of range (3-sigma) among the 14,400 codes. We note that this calibration is not needed for single-shot measurement. After calibration, the TDC achieves a DNL of 1.41 LSB (maximum) and 0.28 LSB_rms_. The INL performance is 2.31 LSB (maximum) and 0.96 LSB_rms_. Before calibration, we obtain a DNL of 0.31 LSB_rms_ and an INL of 1.41 LSB_rms_. When the INL is used to estimate the discrepancy between the estimated and real distance, the result corresponds to an error of 5.42 cm. 

In cyclic TDCs based on an oscillator, nonlinearity occurs due to layout mismatch, supply noise, signal cross-talk, and PVT variation. Compared to other studies (see Table 1), the proposed TDC achieves good linearity. We note that the shape of INL is changed periodically. This is attributed to the accumulated nonlinearity in the coarse step. Using the measured data, we obtain the number of bits *N*_Bit_ = log_2_ (maximum input range/resolution) to be 9.88 bits. Taking nonlinearity into consideration, we obtain the equivalent number of linear bits *N*_linear_ = *N*_Bit_ − log_2_(INL + 1) to be 8.15 bits [23].

**Figure 18:**
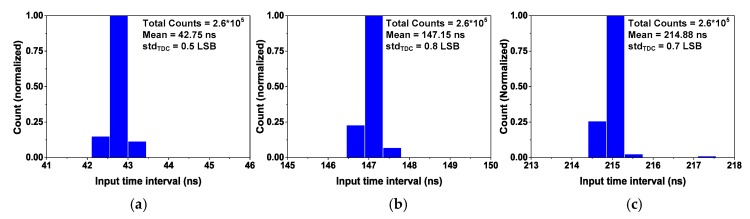
Single-shot measurement. (**a**) *T*_input_ = 41.2 ns, (**b**) 148.3 ns, (**c**) 217.7 ns.

The precision of the TDC is evaluated using the single-shot measurement. Figure 18 shows the result for three cases of *T*_input_. In each measurement, we keep the same *T*_input_ and repeat the conversion up to 260,000 times, which is large enough to observe the precision of the TDC. The result shows a standard deviation, std_TDC_, of 0.5, 0.8, and 0.7 LSB for *T*_input_ = 41.2, 148.3, and 217.7 ns, respectively. The results agree with our expectation that std_TDC_ increases with *T*_input_ due to accumulated jitter. 

Table 1 shows the performance comparison with other studies. The work in [5] integrates TDCs for single-photon avalanche diode array to perform time-correlated single-photon counting. By using an external delay-locked loop (DLL), this work reduces the circuit complexity; the drawback is relatively poor linearity. The work in [6] presents a gated-Vernier oscillator that achieves a resolution of 7.3 ps with a relatively small input range of 9 ns. Using a look-up table for correcting the INL error, this work achieves a DNL of 0.8 LSB_rms_ and an INL of 1.2 LSB_rms_. The work in [13] presents a detailed analysis of compensating PVT variations for the TDCs. By using a global compensation loop based on a phase-locked loop (PLL), the TDC in this work achieves an input range of 716 ns with a resolution of 357 ps. The work in [21] realizes a cyclic TDC using a hardware description language (HDL) which allows chip synthesis with automatic place-and-route tools. The work in [23] presents a two-step TDC based on a pseudo-differential TVC and a 6-bit SAR ADC. This work achieves a high conversion rate of 120 MS/s with a power of 3.73 mW. These works achieve a high resolution of 5.5 ps [21] and 0.63 ps [23]. However, the input range is 4.5 ns and 0.3 ns, which is not sufficient for TOF applications that demand wide ranges. Another work in [31] presents per-pixel TDC for PET imaging. The result achieves a resolution of 64.5 ps with an input range of 50 ns, however, its relatively poor linearity (max INL = 3.9 LSB) needs further correction. Thus, this work can suffer from a large error when applied to a TOF range sensor. The work in [32] presents detailed circuit techniques for all digital TDC to achieve a high resolution of 10 ps, however, only a limited amount of measured data is available. Two works developed for TOF imager, which realizes the TDC using a ring oscillator and a ripple counter, present TDCs achieving a high input range [13,33]. By locking phase and frequency among the ring oscillators, the TDC in [33] achieves an input range of 2 μs with a resolution of 125 ps. With a simple structure, it is suitable for realizing in-pixel TDCs for dTOF image sensors; compared to our work, the linearity performance (max INL = LSB) needs further improvement.

The results in Table 1 show that it is challenging to achieve both high dynamic range and high resolution with a good linearity. Those TDCs that target PLL applications achieve a high resolution with a good figure-of-merit (FOM), however, provide only a limited input range [21,23]. The systems developed for TOF applications provide an increased input range [5,32], but show relatively poor linearity. References [21,23] achieve better FOM than ours, but these works are realized using the 65 nm process for a small input range of 4.5 and 0.3 ns, respectively. Obviously, the range is not suitable for TOF applications requiring a high input range. Except for [13,33], which are realized using a simple ring oscillator and a ripple counter, our work achieves the lowest power consumption. In the cyclic TDC, a large part of power is consumed by DCOs. Therefore, we carefully design the DCOs for low power: (1) we use the RSTD signal to stop the DCO when the conversion is done; (2) we use a small number of five ring stages; (3) we operate the DCOs in low speed although this approach trades the resolution for power. Overall, our TDC in this work achieves a relatively high input range of 355 ns with a good linearity and a low power.

The number of bits *N*_Bit_ can be used to quantify the dynamic range by considering input range and the resolution. A better metric is *N*_linear_ which takes the linearity performance into account. Compared to other work [23,31], our work achieves an *N*_linear_ of 8.15 which indicates good linearity. Increasing performance by 1-bit in an ADC with more than 12-bit resolution is challenging when both area and linearity is constrained. Even though the TDC in this study doesn’t use a capacitor as do SAR ADC, the area and circuit complexity increase in a similar manner. Therefore, a TDC achieving both high dynamic range and resolution tends to be costly and power-hungry, which will be challenging to realize in portable applications. The proposed TDC is suitable for low-power TOF sensor applications demanding both a high input range and a good linearity. 

## 4. Conclusions

In this paper, a low-power cyclic Vernier two-step TDC having a high input range has been designed, fabricated, and successfully characterized. The high input range is achieved by addressing the nonlinearity caused by the startup time in the conventional cyclic TDC. We solve this problem successfully by using DCOs with matched startup times and a high-precision alignment detector. The alignment detector performs precise detection of edge alignment of the two DCOs and controls the counters with proper timing to compensate for the delay caused by the startup time. The proposed TDC achieves a high input range up to 355 ns and a resolution of 377 ps while consuming an average power of 0.65 mW. The proposed TDC can find a useful application in various TOF applications such as TOF ranging and biomedical imaging. 

## Figures and Tables

**Table 1:** Performance summary and comparison.

	[5]	[6]	[13]	[21]	[23]	[31]	[33]	This Work
Tech. (nm)	130	130	180	65	65	130	65	180
Area (mm^2^)	-	0.03	<0.002	0.006	0.064	-	<0.001	0.028
Type	DLL based	Gated Vernier	Ring oscillator	Cyclic Vernier	TVC, SAR	Ring oscillator	Ring oscillator	Cyclic Vernier
Input range (ns)	64	9	716	4.5	0.3	50 *	2000	355
Resolution (ps)	62.5	7.3	357	5.5	0.63	64.5	125	377
Precision (LSB)	-	2.35	**-**	0.78	1.12	-	-	0.8
DNL (LSB_rms_)	<4	0.8	0.35	-	-	0.28	<2	0.28
INL (LSB_rms_)	<8	1.2	1.5	-	-	-	-	0.96
Max. INL (LSB)	8	2.6	**-**	1	3.0	3.9	3	2.3
Conv. Rate (MS/s)	0.4	1	0.5	10	120	-	-	0.67
Power (mW)	-	1.2	<0.01	2.0 ^†^	3.73	1.19	0.5	0.65
*N*_linear_ ** (bit)	-	8.42	-	8.68	6.89	7.31	-	8.15
FOM ***	-	3.5	-	0.49	0.26	-	-	3.4

* An input range up to 261 ns was reported, but linearity was up to 50 ns. ** Equivalent number of linear bits *N*_linear_ = *N*_Bit_ − log_2_(INL + 1). *** FOM = Power/(2*^N^*^linear^ × Conv. rate) [pJ/conv.-step]. ^†^ This is the maximum power reported in the paper. TVC: time-to-voltage converter; SAR: successive approximation register; DNL: differential nonlinearity; FOM: figure of merit.

## References

[1] Zhang M., Wang H., Liu Y. (2017). A 7.4 ps FPGA-based TDC with a 1024-unit measurement matrix. Sensors.

[2] Perenzoni M., Perenzoni D., Stoppa D. (2017). A 64 × 64-Pixels digital silicon photomultiplier direct TOF sensor with 100-MPhotons/s/pixel background rejection and imaging/altimeter mode with 0.14% precision up to 6 km for spacecraft navigation and landing. IEEE J. Solid-State Circuits.

[3] Tanveer M., Nissinen I., Nissinen J., Kostamovaara J., Borg J., Johansson J. Time-to-digital converter based on analog time expansion for the 3D time of flight cameras. Proceedings of the Image Sensors and Imaging Systems 2014.

[4] Min S.D., Kim J.K., Shin H.S., Yun Y.H., Lee C.K., Lee M. (2010). Noncontact respiration rate measurement system using an ultrasonic proximity sensor. IEEE Sens. J..

[5] Field R.M., Realov S., Shepard K.L. (2014). A 100 fps, time-correlated single-photon-counting-based fluorescence-lifetime imager in 130 nm CMOS. IEEE J. Solid-State Circuits.

[6] Cheng Z., Deen M.J., Peng H. (2016). A low-power gateable Vernier ring oscillator time-to-digital converter for biomedical imaging applications. IEEE Trans. Biomed. Circuits Syst..

[7] Paschalidis N., Stamatopoulos N., Karadamoglou K., Kottaras G., Paschalidis V., Sarris E., McNutt R., Mitchell D., McEntire R. (2002). A CMOS time-of-flight system-on-a-chip for spacecraft instruments. IEEE Trans. Nucl. Sci..

[8] Gariepy G., Tonolini F., Henderson R., Leach J., Faccio D. (2016). Detection and tracking of moving objects hidden from view. Nat. Photonics.

[9] Poland S.P., Krstajić N., Monypenny J., Coelho S., Tyndall D., Walker R.J., Devauges V., Richardson J., Dutton N., Barber P. (2015). A high speed multifocal multiphoton fluorescence lifetime imaging microscope for live-cell FRET imaging. Biomed. Opt. Express.

[10] O’Toole M., Lindell D.B., Wetzstein G. (2018). Confocal non-line-of-sight imaging based on the light-cone transform. Nature.

[11] Shin D., Xu F., Venkatraman D., Lussana R., Villa F., Zappa F., Goyal V.K., Wong F.N.C., Shapiro J.H. (2016). Photon-efficient imaging with a single-photon camera. Nat. Commun..

[12] Ren X., Connolly P.W.R., Halimi A., Altmann Y., McLaughlin S., Gyongy I., Henderson R.K., Buller G.S. (2018). High-resolution depth profiling using a range-gated CMOS SPAD quanta image sensor. Opt. Express.

[13] Vornicu I., Carmona-Galán R., Rodríguez-Vázquez Á. (2017). Compensation of PVT variations in ToF imagers with in-pixel TDC. Sensors.

[14] Gao Z., Yang C., Xu J., Nie K. (2015). A dynamic range enhanced readout technique with a two-step TDC for high speed linear CMOS image sensors. Sensors.

[15] Xu Z., Lee S., Miyahara M., Matsuzawa A. (2016). A 3.6 GHz low-noise fractional-N digital PLL using SAR-ADC-based TDC. IEEE J. Solid-State Circuits.

[16] Vercesi L., Liscidini A., Castello R. (2010). Two-dimensions Vernier time-to-digital converter. IEEE J. Solid-State Circuits.

[17] Intermite G., McCarthy A., Warburton R.E., Ren X., Villa F., Lussana R., Waddie A.J., Taghizadeh M.R., Tosi A., Zappa F. (2015). Fill-factor improvement of Si CMOS single-photon avalanche diode detector arrays by integration of diffractive microlens arrays. Opt. Express.

[18] Pavia J.M., Wolf M., Charbon E. (2014). Measurement and modeling of microlenses fabricated on single-photon avalanche diode arrays for fill factor recovery. Opt. Express.

[19] Sun P., Charbon E., Ishihara R. (2014). Flexible ultrathin-body single-photon avalanche diode with dual-side illumination. IEEE J. Sel. Top. Quantum Electron..

[20] Kim Y., Kim T.K. (2014). An 11b 7 ps resolution two-step time-to-digital converter with 3-D Vernier space. IEEE Trans. Circuits Syst. I-Regul. Papers.

[21] Park Y., Wentzloff D.D. (2011). A cyclic vernier TDC for ADPLLs synthesized from a standard cell library. IEEE Trans. Circuits Syst. I-Regul. Papers.

[22] Lu P., Wu Y., Andreani P. (2016). A 2.2-ps two-dimensional gated-vernier time-to-digital converter with digital calibration. IEEE Trans. Circuits Syst. II-Exp. Briefs.

[23] Kim J., Kim Y.H., Kim K.S., Yu W., Cho S.H. (2015). A hybrid-domain two-step time-to-digital converter using a switch-based time-to-voltage converter and SAR ADC. IEEE Trans. Circuits Syst. II-Exp. Briefs.

[24] Andersson N.U., Vesterbacka M. (2014). A Vernier time-to-digital converter with delay latch chain architecture. IEEE Trans. Circuits Syst. II-Exp. Briefs.

[25] Keränen P., Kostamovaara J. (2015). A wide range, 4.2 ps (rms) precision cmos TDC with cyclic interpolators based on switched-frequency ring oscillators. IEEE Trans. Circuits Syst. I-Regul. Papers.

[26] Nutt R. (1968). Digital time intervalometer. Rev. Sci. Instrum..

[27] Nguyen V.N., Lee J.-W. A low power two-step cyclic time-to-digital converter without startup time error in 180 nm CMOS. Proceedings of the 2018 2nd International Conference on Recent Advances in Signal Processing, Telecommunications & Computing.

[28] Wu J., Jiang Q., Song K., Zheng L., Sun D., Sun W. (2017). Implementation of a high-precision and wide-range TDC with three-level conversion scheme. IEEE Trans. Circuits Syst. II-Exp. Briefs.

[29] Baker R.J. (2010). The Inverter. CMOS Circuit Design, Layout, and Simulation.

[30] Henzler S. (2010). Theory of TDC Operation. Time-to-Digital Converter.

[31] Braga L.H.C., Gasparini L., Grant L., Henderson R.K., Massari N., Perenzoni M., Stoppa D., Walker R. (2014). A fully digital 8 × 16 SiPM array for PET applications with per-pixel TDCs and real-time energy output. IEEE J. Solid-State Circuits.

[32] Huang H.Y., Hung W.C., Cheng H.W., Lu C.H. (2011). All digital time-to-digital converter with high resolution and wide detect range. Eng. Lett..

[33] Ximenes A.R., Padmanabhan P., Charbon E. (2018). Mutually coupled time-to-digital converters (TDCs) for direct time-of-flight (dTOF) image sensors. Sensors.

